# Citrus Flavanone Narirutin, In Vitro and In Silico Mechanistic Antidiabetic Potential

**DOI:** 10.3390/pharmaceutics13111818

**Published:** 2021-10-31

**Authors:** Ashraf Ahmed Qurtam, Hamza Mechchate, Imane Es-safi, Mohammed Al-zharani, Fahd A. Nasr, Omar M. Noman, Mohammed Aleissa, Hamada Imtara, Abdulmalik M. Aleissa, Mohamed Bouhrim, Ali S. Alqahtani

**Affiliations:** 1 Biology Department, College of Science, Imam Mohammad Ibn Saud Islamic University (IMSIU), Riyadh 11623, Saudi Arabia; AAQURTAM@imamu.edu.sa (A.A.Q.); MMyAlzahrani@imamu.edu.sa (M.A.-z.); msaleissa@imamu.edu.sa (M.A.); 2 Laboratory of Biotechnology, Environment, Agrifood and Health, Faculty of Sciences Dhar El Mahraz, University of Sidi Mohamed Ben Abdellah, Fez 30000, Morocco; Hamza.mechchate@usmba.ac.ma; 3 Department of Pharmacognosy, College of Pharmacy, King Saud University, Riyadh 11451, Saudi Arabia; fnasr@ksu.edu.sa (F.A.N.); onoman@ksu.edu.sa (O.M.N.); alalqahtani@ksu.edu.sa (A.S.A.); 4 Faculty of Arts and Sciences, Arab American University Palestine, Jenin 240, Palestine; Hamada.tarayrah@gmail.com; 5 King Khaled Eye Specialist Hospital (KKESH), Riyadh 11462, Saudi Arabia; aaleissa@kkesh.med.sa; 6 Laboratory of Bioresources, Biotechnology, Ethnopharmacology and Health, Faculty of Sciences, Mohammed First University, Oujda B.P. 717, Morocco; mohamed.bouhrim@gmail.com

**Keywords:** narirutin, naringenin rutinoside, isonaringin, molecular docking, mechanism of action, enzyme, receptors

## Abstract

Citrus fruits and juices have been studied extensively for their potential involvement in the prevention of various diseases. Flavanones, the characteristic polyphenols of citrus species, are the primarily compounds responsible for these studied health benefits. Using in silico and in vitro methods, we are exploring the possible antidiabetic action of narirutin, a flavanone family member. The goal of the in silico research was to anticipate how narirutin would interact with eight distinct receptors implicated in diabetes control and complications, namely, dipeptidyl-peptidase 4 (DPP4), protein tyrosine phosphatase 1B (PTP1B), free fatty acid receptor 1 (FFAR1), aldose reductase (AldR), glycogen phosphorylase (GP), alpha-amylase (AAM), peroxisome proliferator-activated receptor gamma (PPAR-γ), alpha-glucosidase (AGL), while the in vitro study looked into narirutin’s possible inhibitory impact on alpha-amylase and alpha-glucosidase. The results indicate that the studied citrus flavanone interacted remarkably with most of the receptors and had an excellent inhibitory activity during the in vitro tests suggesting its potent role among the different constituent of the citrus compounds in the management of diabetes and also its complications.

## 1. Introduction

Diabetes is a severe, long-term disease that has a significant effect on the lives and well-being of people, families, and communities all over the globe. It is among the top ten causes of mortality in adults, with an estimated four million fatalities worldwide in 2017 [1]. The International Diabetes Federation (IDF) has been tracking diabetes on national, regional, and worldwide scales since 2000. In 2009, 285 million individuals were projected to have diabetes (including T1D and T2D), 366 million in 2011, 382 million in 2013, 415 million in 2015, 425 million in 2017, 463 million in 2019 and the global projection is 578 million by 2030, and 700 million by 2045 [1,2,3,4,5]. Diabetes has a tremendous social cost in terms of increased medical expenses, lost productivity, early death, and intangible costs such as decreased quality of life. Diabetes cost the global health system 727 billion USD in 2017 [1]. Furthermore, changes in the demography of the diabetic community, health-care use and delivery patterns, technology, medical expenses, insurance coverage, and economic circumstances continue to have an impact on the economic burden of diabetes [6]. While several synthetic oral medicines have been established to treat diabetes, it is still a struggle to control diabetes without any side effects [7]. A new surge of scientific interest in conventional practices has been stimulated by increased research about complementary drugs and natural therapies, and the desire to look for more powerful agents with less side effects [8,9,10,11]. It is commonly known that low intake of unhealthy foods, daily physical exercise and strong consumption of plant-based products help to maintain a stable state of health [12]. In fact, an elevated dietary amount of fruit and vegetables is correlated with a decreased risk of some life-threatening diseases, such as cardiovascular disease and cancer [13]. Many phenolic secondary metabolites (flavonoids) found in plant-derived foods are now increasingly recognized to have beneficial health effects on the prevention and treatment of diseases [14,15]. Flavonoids are plant secondary metabolites belonging to polyphenolic family, and constitute a very interesting and important class of natural products commonly present in fruits, vegetables and some beverages [16]. They are an essential component of a variety of nutraceuticals, pharmaceutical, medicinal and cosmetic uses due to their broad spectrum of health-promoting properties [17,18]. This is due to their ability to regulate critical cellular enzyme activities, as well as their antioxidative, anti-inflammatory, antimutagenic, and anticarcinogenic characteristics [19,20]. They are phenolic chemicals of low molecular weight and one of the most distinctive groups of chemicals found in higher plants.

Flavanones, which are found in all citrus fruits such as oranges, lemons and grapes, are one of the most significant subgroups of flavonoids [21,22]. Because of their free radical-scavenging characteristics, they are linked to a variety of health advantages. Citrus fruit juice and peel contain these chemicals, which give them a bitter flavor. Citrus flavonoids have pharmacological actions against reactive oxygen species, inflammation, and are involved in lowering blood lipids and cholesterol [23]. Different studies have investigated their role (i.e., luteolin, apigenin) in epigenetic therapy and cell gene expression by regulating HDAC1, HDAC2 [24] and MMP-2 [25].

**Figure 1:**
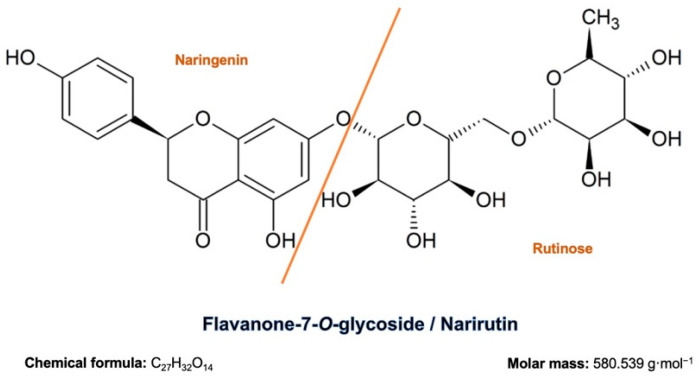
Narirutin chemical structure, formula and molar mass.

In this study, we looked into the antidiabetic potential of narirutin (flavanone-7-O-glycoside), a flavanone family representative, consisting of the flavanone naringenin bonded with the disaccharide rutinose (Figure 1), through different approaches to assess its mechanistic proprieties as a preventive and curative natural alternative. Molecular docking was used to evaluate molecular interaction with the following receptors: dipeptidyl-peptidase 4 (DPP4), protein tyrosine phosphatase 1B (PTP1B), free fatty acid receptor 1 (FFAR1), aldose reductase (AldR), glycogen phosphorylase (GP), alpha-amylase (AAM), peroxisome proliferator-activated receptor gamma (PPAR-γ), and alpha-glucosidase (AGL). The alpha-amylase and alpha-glucosidase enzymes were tested in vitro to see whether the compound has any inhibitory action.

## 2. Results and Discussion

### 2.1. Molecular Docking

To confirm the interaction of the receptor with the ligand, literature screening was performed to identify the active site of the receptor and the amino acids composing it. Table 1 shows the result of the in silico simulation of the studied molecule and the selected receptors (affinity), the active site description and the amino acids that interact with a hydrogen bond in the site.

#### 2.1.1. PTP1B

Resistance to insulin’s cellular action, a basic pathophysiological flaw associated with the global obesity pandemic, is linked to the development of type 2 diabetes and metabolic syndrome, a collection of cardiovascular risk factors. The discovery of new pharmacological medicines that help alleviate insulin resistance may be crucial not only for the prevention and treatment of diabetes but also for lowering the cardiovascular risk profile associated with it [44,45].

Although that PTP1B direct action to control the cardiovascular function is still unclear, there is more evidence linking this receptor with the inhibition of the regulation of metabolic function and cardiovascular function because of its direct interaction with various receptor tyrosine kinase signaling pathways [46]. Overall, these investigations have opened the way for the commercialization of PTP1B inhibitors, which may be used as a new kind of “insulin sensitizer” in the treatment of type 2 diabetes and cardiovascular/metabolic syndromes [47].

**Figure 2:**
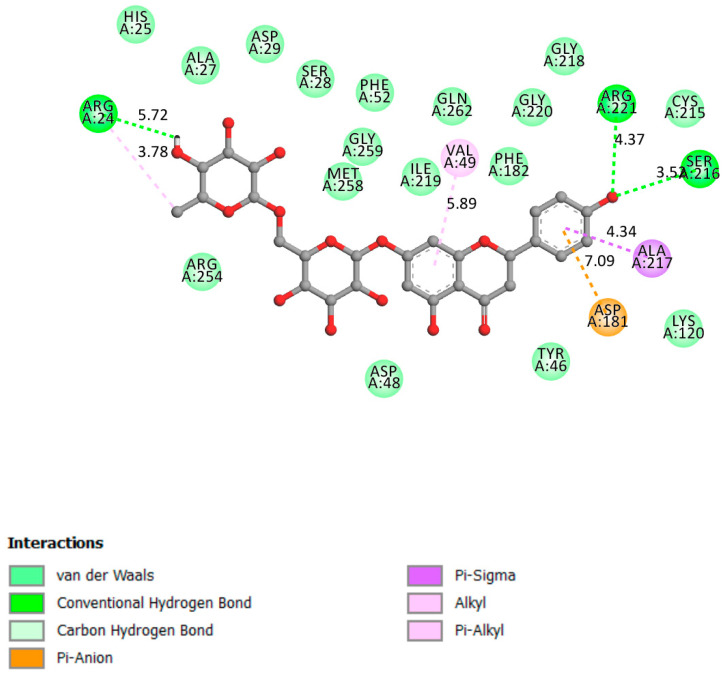
Two-dimensional scheme of the narirutin interactions with PTP1B.

Figure 2 shows that narirutin had a strong contact with the catalytic site and the active site, with a docking affinity of 8.5 kcal/mol and three hydrogen bonds formed.

#### 2.1.2. DPP4

DPP4 inhibitors have been linked to better blood glucose management and lower fasting and postprandial blood glucose levels while avoiding weight gain [48]. DPP4 inhibition by a chemotherapeutic agent may raise the levels of circulating endogenous GLP-1 by prolonging its half-life, thus increasing GLP-1’s beneficial effects in glucose-dependent insulin production and cell restoration. DPP-4 belongs to the serine protease family, which also includes the fibroblast activation protein (FAP), DPP-8, and DPP-9 [49]. DPP-4 inhibitors provide many benefits, including a reduced risk of hypoglycemia, minimal weight gain, and the possibility for pancreatic -cell repair and separation [50].

**Figure 3:**
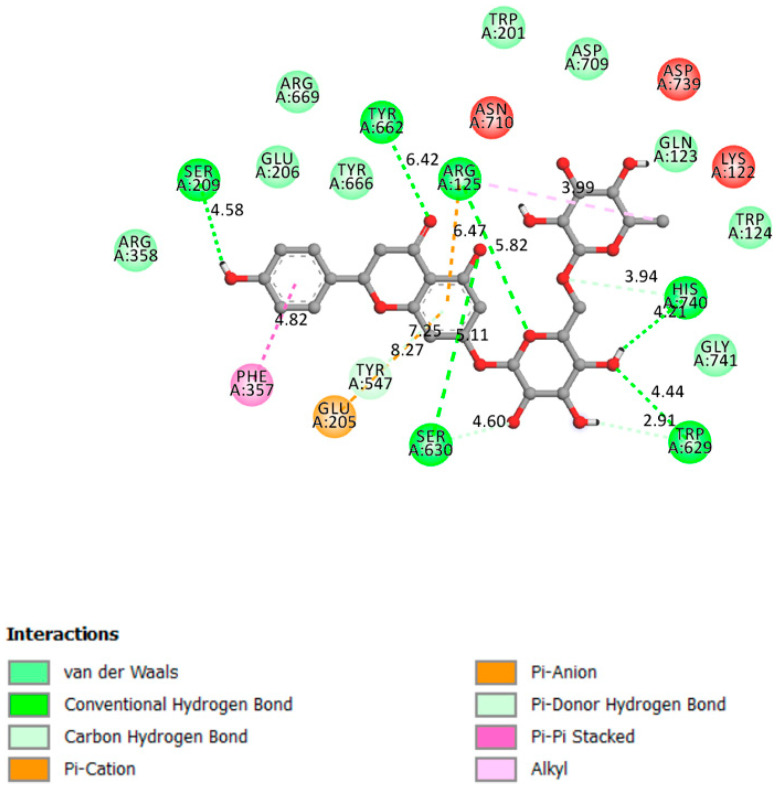
Two-dimensional scheme of the narirutin interactions with DPP4.

In the α/β-hydrolase domain, excellent interactions were established between narirutin and DPP4 receptor, with a docking affinity of 10.4 kcal/mol and 6 hydrogen bonds formed, as shown in Figure 3.

#### 2.1.3. FFAR1

Free fatty acid receptor 1 (FFAR1) agonists have recently been discovered as a promising anti-diabetic target, since they regulate secretion stimulated by glucose in the pancreatic cells without causing hypoglycemia [51]. The FFAR1 mode of action is via interaction with a G protein. The coupling mechanism activates phospholipase-C, which regulates inositol triphosphate and the endoplasmic reticulum’s release of intracellular Ca^2+^. As a result, insulin secretion improves in a glucose concentration-dependent manner [52].

**Figure 4:**
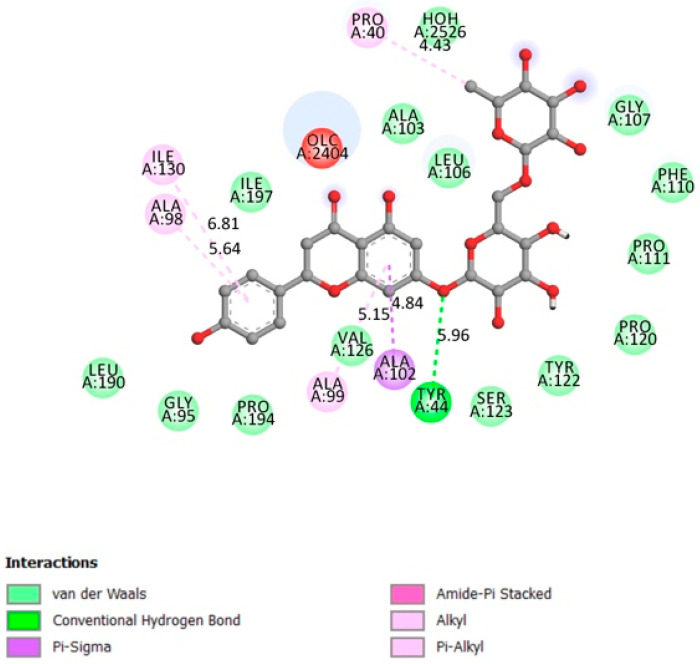
Two-dimensional scheme of the narirutin interactions with FFAR1.

The Docking results demonstrated good interactions between narirutin and the FFAR1 receptor (Affinity of −8.3 kcal/mol) with one single hydrogen bond formed (Figure 4).

#### 2.1.4. Alpha-Amylase

Alpha-amylases are present in a broad range of living creatures (plants, animals, bacteria, and fungi). They are also present in human salivary glands, and the pancreas secretes them into the small intestine on a regular basis during digestion. They belong to the amylase family of enzymes whose main role in the body is to catalyze the hydrolysis of complex polysaccharides into small digestible mono, di and trisaccharide, converted afterwards to glucose as the fuel for energy production [53,54].

**Figure 5:**
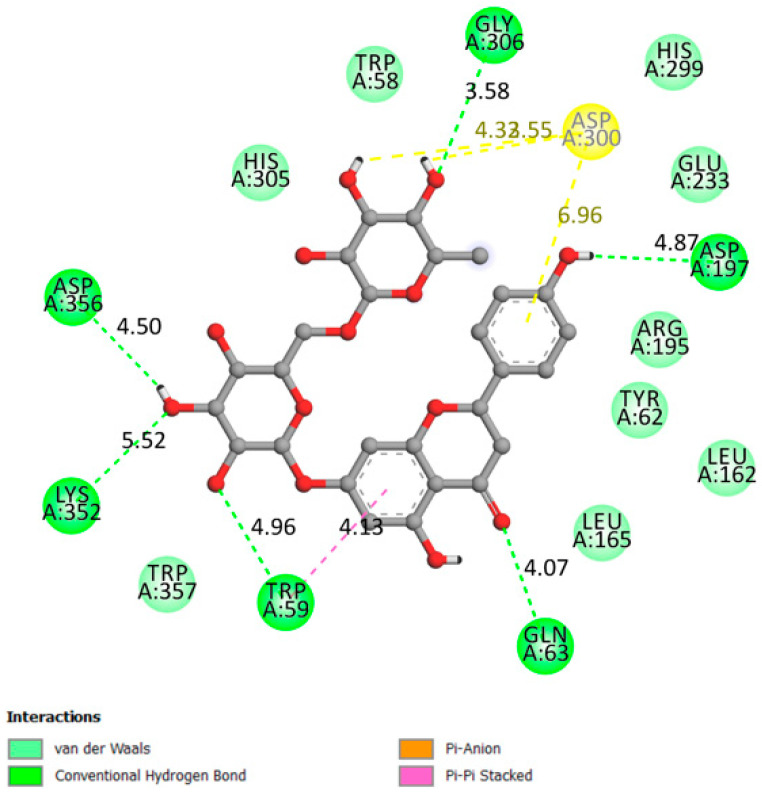
Two-dimensional scheme of the narirutin interactions with AAM.

Figure 5 shows the interactions between narirutin with the alpha-amylase receptor on the catalytic residues (affinity of −9.9 kcal/mol) with the formation of six hydrogen bonds.

#### 2.1.5. PPARγ

Peroxisome proliferator-activated receptor gamma (PPARγ) is a protein that controls gene expression and is found in key reproductive components [55]. PPARs are important regulators of cellular differentiation, cellular growth, and carbohydrate, lipid, and protein metabolism in humans. PPARγ has been linked to a key role in glucose homeostasis [56], making it a promising target for T2DM treatment. It is highly expressed in adipose tissue and, for optimum DNA binding and transcriptional activity, needs heterodimerization with the retinoid X-receptor (RXR) [56]. PPARγ stimulates the expression of glucose transporters (GLUT4) and subsequent translocation, leading to decreased levels of blood glucose, blood gluconeogenesis repression and enhanced lipide storage and glucose intake into muscles, and performs a number of psychological activities [57]. Increased insulin sensitizing effect occurs when adipose genes are activated.

Docking findings and various poses analysis revealed no established interaction between narirutin and the PPARγ receptor. The results were confirmed when compared with the default ligand cocrystalized with the receptor PDB format (Rosiglitazone), which binds perfectly into the receptor active site.

#### 2.1.6. Alpha-Glucosidase

Alpha-glucosidase is one of the digestive enzymes, its main function is to accelerate the hydrolysis of polysaccharides (starch) to glucose (acts on (14) bonds) in order to promote glucose absorption and, therefore, raise blood glucose levels [58]. This helps to prevent hyperglycemia and maintain normal blood sugar levels by slowing down the digestion of starch and extra dietary carbohydrates [58].

**Figure 6:**
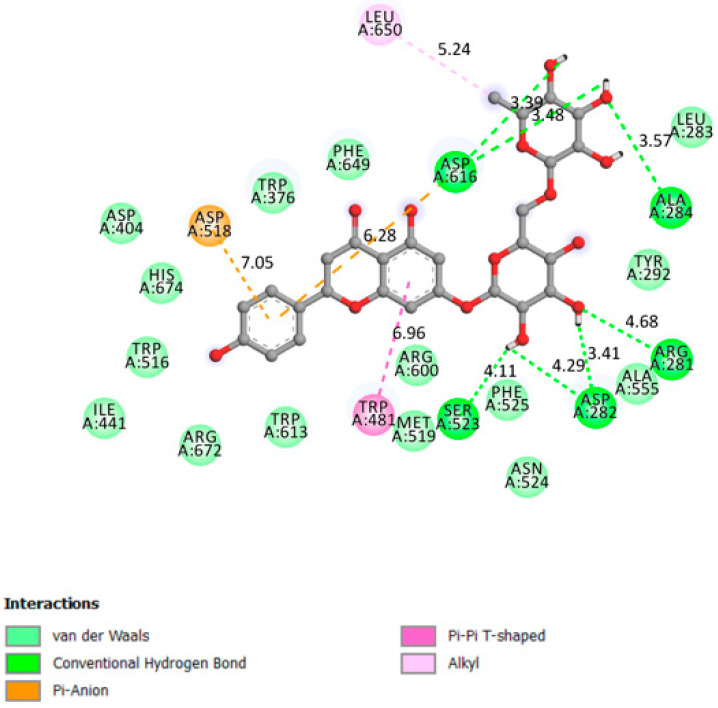
Two-dimensional scheme of the narirutin interactions with AGL.

Figure 6 indicates that narirutin had a good interaction with the receptor active site with 4 hydrogen bonds formed and an affinity of −8.7 kcal/mol.

#### 2.1.7. Aldose Reductase

Aldose reductase is an important enzyme that catalyzes the NAD (P) H-dependent reduction of glucose to sorbitol through the sorbitol-aldose reductase pathway. It is found in numerous organs throughout the body, including red blood cells, retina, Schwann cells, and others. As a consequence of it action, intracellular reactive oxygen species (ROS) accumulate excessively in different organs [59], leading to various health complications [60]. Excessive sorbitol buildup in tissues occurs in T2DM patients with poor sorbitol penetration and metabolism, leading to DM-related problems such as cataracts and glaucoma.

As a result, aldose reductase inhibition has been proposed as a possible treatment for T2DM problems.

**Figure 7:**
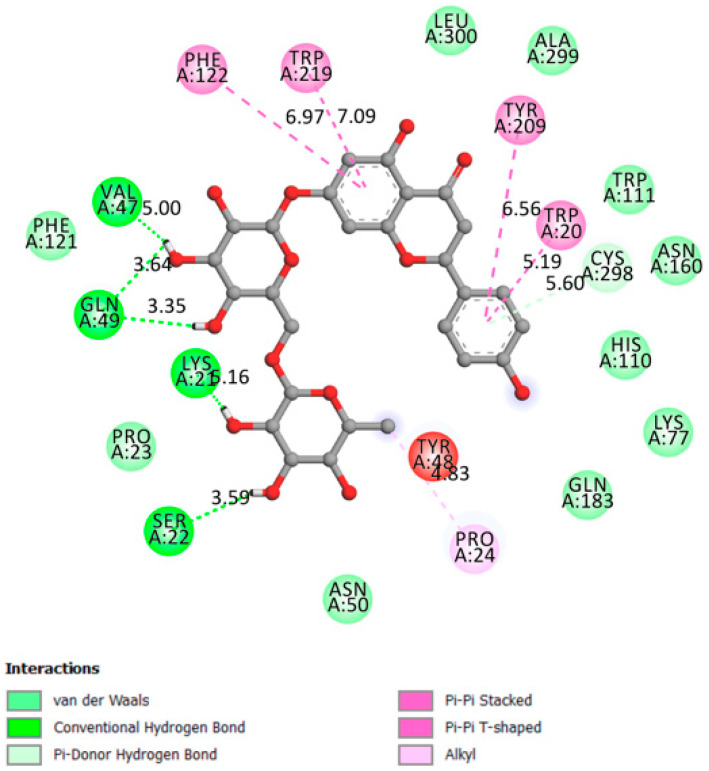
Two-dimensional scheme of the narirutin interactions with AldR.

The interaction between narirutin and AldR demonstrated one of the best affinities (−9.3 kcal/mol) with the ligand fitting right into the receptor barrel core (active site [41]) with five hydrogen bonds formed (Figure 7).

#### 2.1.8. Glycogen Phosphorylase

Glycogen phosphorylase catalyzes glycogen hydrolysis to produce glucose-1-phosphate. Allosteric effectors and reversible phosphorylation regulate the activity of GP [61]. Phosphorylation control is a widespread intracellular mechanism that regulates, controls, and participates in signal transmission [61]. The liver may store glucose as glycogen and then generate and release glucose into the bloodstream through a reverse mechanism. As a result, the liver is closely connected to glycemic regulation, and hepatic metabolism provides numerous therapeutic options in the setting of T2DM [62].

**Figure 8:**
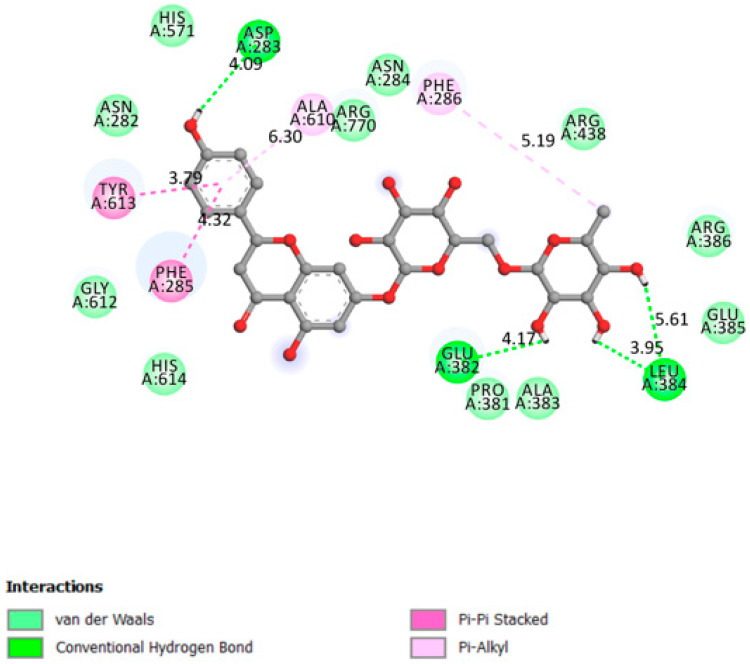
Two-dimensional scheme of the narirutin interactions with Glycogen Phosphorylase receptor.

Figure 8 displays the interaction of narirutin with GP’s 280’s loop (active site), with an affinity of −8.3 kcal/mol and three hydrogen bonds formed.

The excellent affinities of the receptors/ligand interactions ranged between −8.3 and −10.4 kcal/mol, and narirutin interaction with all receptors demonstrate that this molecule can act as a multitarget medicine to treat diabetes, targeting multiple receptors involved directly and indirectly in overall diabetic status. The in silico study concluded that the predicted mechanism of action exhibited by Narirutin is the inhibition of PTP1B, DPP4, AAM, AGL, AldR, GP, and the activation of FFAR1.

To have a better insight concerning the obtained results, we compared the obtained results with those obtained with other molecules such as gentisic acid [8] and amentoflavone [63] using the same methodology and against similar receptors. Gentisic acid demonstrated poor affinities ranging between −5.6 and −6.9 kcal/mol, which were partially confirmed with an in vitro investigation in which the molecule also demonstrated low activity. On the other hand, amentoflavone showed excellent affinities ranging between −8.8 and −11.3 kcal/mol, which was in total accordance with the molecule being well known with antidiabetic potential and tested in vivo [64].

Narirutin in vitro testing was done to partially confirm the results obtained above.

### 2.2. In Vitro Assays

One of the most effective methods for treating diabetes is to prevent glucose absorption. By blocking the digestive enzymes that hydrolyze polysaccharides into small absorbable pieces, postprandial high blood glucose is avoided. Alpha-glucosidase and alpha-amylase are two of these enzymes. The inhibitory impact of narirutin on these two enzymes were investigated to identify one or more of this plant’s mechanisms of action.

#### 2.2.1. Alpha-Amylase Inhibitory Effect

Alpha-amylase is considered as one of the most essential enzymes in the digestive process [34]. because of its critical involvement in the breakdown of polysaccharides. Saliva and pancreatic juice are the two most common places to find it. One approach for avoiding increasing postprandial blood glucose is to target and inhibit this enzyme [39].

**Figure 9:**
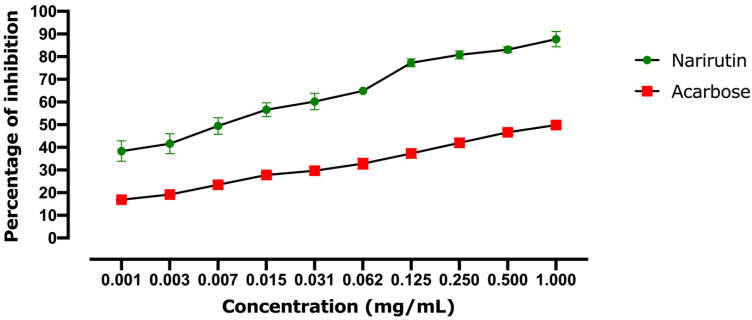
Alpha-amylase inhibitory effect results. Values are expressed as mean ± SD (*n* = 3).

Figure 9 shows the ability of narirutin to inhibit alpha-amylase. Since the concentration of narirutin obviously affects the quantity of enzyme inhibited, the inhibition of the enzyme seems to be linked to the dose. The estimated IC50 revealed that acarbose (positive control) had a lower inhibitory potential than narirutin, with an IC50 of 1.012 mg/mL for acarbose compared to 0.0066 mg/mL for narirutin. In comparison to acarbose, narirutin showed outstanding activity, which was consistent with the in silico findings.

#### 2.2.2. Alpha-Glucosidase Inhibitory Effect

One of the major digesting enzymes is alpha-glucosidase, which is located in the mucosal brush border of the small intestine. Its job is to break down and convert complicated carbohydrates into short, fast, and absorbable sugars. Inhibition is a key strategy for slowing glucose absorption and avoiding high postprandial blood glucose levels, both of which may delay the onset of diabetes.

**Figure 10:**
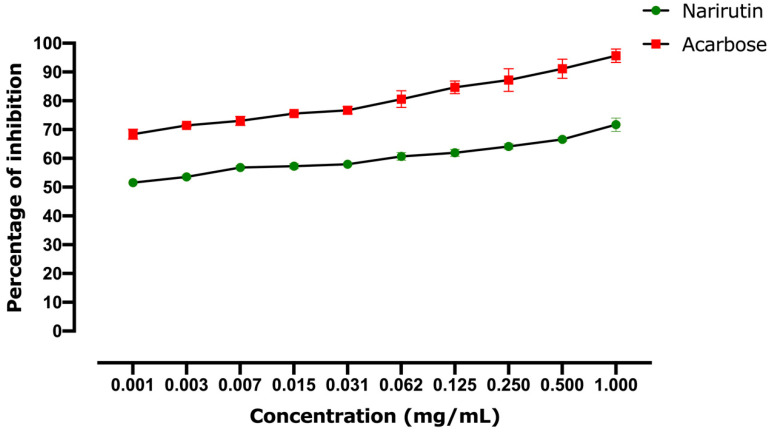
Alpha-glucosidase inhibitory effect results. Values are expressed as mean ± SD (*n* = 3).

Figure 10 shows narirutin’s ability to block alpha-glucosidase. The inhibitory impact is linked to the narirutin concentration since the highest concentrations showed the greatest inhibition activity. The estimated IC50 revealed that acarbose has a more powerful inhibitory action (IC50 = 0.00035 mg/mL) than that observed with narirutin (IC50 = 0.00091 mg/mL). Nevertheless, the obtained results for narirutin are still considered powerful, and consistent with the docking findings.

As can be seen from Table 2, the results of the in vitro testing correlate perfectly with those obtained in silico. The difference in the affinity score of narirutin and acarbose against alpha-amylase in silico (−9.9 and −8.1 respectively) was also seen in the in vitro test (0.0066 and 1.012, respectively). Against alpha-glucosidase, almost similar results were obtained in both tests, confirming the accuracy of the performed analysis.

## 3. Materials and Methods

### 3.1. Chemicals and Reagents

Chemicals and reagents used in this study were analytical grade (obtained from Sigma Aldrich, St. Louis, MO, USA). (Narirutin CAS: 14259-46-2; MW: 580.53, alpha-amylase CAS: 9000-85-5, alpha-glucosidase CAS: 9001-42-7).

### 3.2. Molecular Docking

#### 3.2.1. Preparation of the Ligand

The SDF format of Narirutin (Figure 1) was obtained from PubChem (CID: 442431). The SDF file was converted to the PDBQT format using AutoDock Tools. For the final ligand preparation Gasteiger partial charges were added, rotatable bonds were defined and the nonpolar hydrogen atoms were merged.

#### 3.2.2. Preparation of the Receptors

Each receptor’s PDB file was obtained from the protein data bank website [65]. The receptors’ X-ray crystal structures were selected for their completeness, resolution, and compatibility with our study goal. Details of the selected receptors are described in Table 3.

For the receptors, Discovery Studio Visualizer v 19.1.0 (BIOVIA, San Diego, CA, USA (windows software)) was used first to begin the preparation of the receptors before running the analysis by deleting water molecules and heteroatoms. AutoDock Tools v1.5.6 (Scripps Research, San Diego, CA, USA) were used later on to add Gasteiger charges and polar hydrogen atoms. The final file was converted to the PDBQT format to prepare for molecular docking.

#### 3.2.3. Simulation

AutoDock Tools were used to establish the grid box size for each receptor, and AutoDock Vina was used to run docking simulations for the narirutin (Ligand) and the eight receptors [66]. The exhaustiveness of the simulation was set to 24. Discovery Studio Visualizer was used to visualize the protein-ligand complexes.

### 3.3. Narirutin In-Vitro Inhibition Potential on Digestive Enzymes

#### 3.3.1. Alpha-Amylase Inhibition Assay

The test was carried out according to Mechchate procedure [67]. A solution of 0.2 mL of 0.5 M Tris–HCl buffer (pH 6.9) containing 0.01 M CaCl_2_ was combined with 2 mg of starch to make the substrate solution. The substrate solution was divided into test tubes, boiled for 5 min, and then preincubated for 5 min at 37 °C. Narirutin was dissolved in DMSO and produced at doses ranging from 1 to 1000 g/mL. Porcine pancreatic amylase (0.1 mL in Tris-HCl buffer (2 units/mL) was added to the test tube holding the substrate solution after the narirutin solution (0.2 mL) was added at various concentrations. The reaction was carried out for 10 min at 37 °C before being stopped by adding 0.5 mL of 50% acetic acid to each test tube. The supernatant optical density was measured using a spectrophotometer at 595 nm after centrifugation (3000 rpm for 5 min at 4 °C). Acarbose was utilized as a positive control (alpha-amylase inhibitor) in this test. For each concentration, the tests were performed three times.

The inhibitory activity of alpha-amylase was determined using the formula:

alpha-amylase inhibitory activity = [(*A* − *B*)/*A*] × 100 (1)

where the absorbance of the negative control (solution with only DMSO) is *A*, whereas that of the sample (Narirutin and acarbose) is *B*.

After evaluating the inhibitory activity of various concentrations against alpha-amylase, the IC50 values for acarbose and narirutin (concentration needed to inhibit 50% of the enzyme) were calculated.

#### 3.3.2. Alpha-Glucosidase Inhibitory Assay

The test was carried out according to the procedure of Pistia Brueggeman and Hollingsworth [68]. A quantity of 50 microliters of narirutin (concentration ranging between 1–1000 g/mL) was made and incubated for 20 min at 37 °C with a solution comprising 10 microliters of alpha-glucosidase 1 U/mL and 125 μL of 0.1 M phosphate buffer (pH 6.8) A solution of 20 μL of 1 M pNPG (substrate) was added to initiate the reaction, which was then incubated for half an hour. To stop the reaction, 50 microliters of 0.1 N Na_2_CO_3_ were added. A spectrophotometer was used to measure the optical density at 405 nm. Acarbose was utilized as a positive control in this test. For each concentration, the tests were performed three times.

The inhibitory activity of alpha-glucosidase was determined using the formula:

Alpha-glucosidase inhibitory activity = [(*A* − *B*)/*A*] × 100 (2)

where the absorbance of the negative control (solution with only DMSO) is *A*, and that of the sample (Narirutin and acarbose) is *B* After evaluating the inhibitory activity of alpha-glucosidase at various concentrations, the IC50 values for acarbose and narirutin (concentration needed to inhibit 50% of alpha-glucosidase) were calculated.

### 3.4. Statistical Analysis

Statistical analysis was performed using GraphPad prism version 7 for Windows (GraphPad Software, CA, USA). In vitro analysis was done in three replicates and presented as mean ± SD.

## 4. Conclusions

The antidiabetic activity of narirutin was remarkable and indicated strong and real potential by inhibition of PTP1B, DPP4, AAM, AGL, AldR, GP, and the activation of FFAR1 with excellent affinities. This conclusion was partially confirmed by in vitro tests, which confirmed the in silico results of the alpha-amylase and alpha-glucosidase experiments. The results were compared to those obtained with other antidiabetic molecules with strong and well-studied mechanism of action. This citrus flavanone deserves further standardization and complete pharmacological study to confirm its overall impact on diabetes prevention, management and also to prevent the complications.

## Figures and Tables

**Table 1:** Summary of narirutin/receptors affinities.

Receptor	Affinity (kcal/mol)	Active Site Described in Literature	Interaction Confirmed with the Active Site	H-Bonds
PTP1B	−8.5	Trp179, Pro180, Asp181 [26], His214, Ser216, Ala217, Gly218, Ile219, Gly220, and Arg221 [27,28] and the active site on Cys 215 (catalytic loop)	Yes	Arg221, Arg24, Ser216
DPP4	−10.4	DPP4 active site (α/β-hydrolase domain) is identified by the residues from 39 to 51 and from 501 to 706 [29,30].	Yes	Tyr662, Ser209, Ser630, Arg125, His740, Trp629,
FFAR1	−8.3	Active site includes Arg183, Arg258 and Tyr2240 [31]. Binding pocket is on Glu172, Arg183, Ser187, Tyr240, Asn241, Asn244, Arg258 and Tyr91 [32,33].	Yes	Tyr44
Alpha amylase	−9.9	Active site:Asp197, Glu233 and Asp300 and other important AA: Arg337, Arg195, Asn298, Phe265, Phe295, His201, Ala307, Gly306, Trp203, Trp284, Trp59, Tyr62, Trp58, His299 and His101 [34,35,36,37].	Yes	Gly306, Asp197, Gln63, Trp69, Lys352, Asp352
PPAR gamma	-	PPARγ ligand-binding domain: Ser289, His323, Tyr473, and His449 [38].	No	
Alpha glucosidase	−8.7	The amino acids involved in the α-Glucosidase activity are Asp404, Asp518, Arg600, Asp616, and His674 Trp376, Ile441, Trp516, Met519, Trp613, and Phe649 Leu405, Trp481, Asp645, and Arg672 [39,40].	Yes	Asp616, Ala284, Arg281, Asp282, Ser523
Aldose reductase	−9.3	The active site is located in the barrel core clearly seen in the 3D structure [41]	Yes	Val47, Gln49, Lys21, Ser32
Glycogen phosphorylase	−8.3	Active site on amino acids 280–288 (The 280’s loop) [42,43].	Yes	Asp283, Glu382, leu384

**Table 2:** Summary of narirutin and acarbose in vitro and in silico activities against alpha-amylase and alpha-glucosidase.

	Alpha-Amylase In Vitro IC50 (mg/mL)	Alpha-Glucosidase In Vitro IC50 (mg/mL)	Alpha-Amylase In Silico Affinity (kcal/mol)	Alpha-Glucosidase In Silico Affinity (kcal/mol)
Narirutin	0.0066	0.00091	−9.9	−8.7
Acarbose	1.012	0.00035	−8.1	−8.4

**Table 3:** Selected receptors parameters.

Receptor	PID	Resolution (Å)	Classification
Protein tyrosine phosphatase 1B (PTP1B)	1c83	1.80	Hydrolase
Glycogen phosphorylase (GP)	1l5q	2.25	Transferase
Free fatty acid receptor 1 (FFAR1)	4phu	2.33	Hydrolase
Peroxisome proliferator-activated receptor gamma (PPAR gamma)	5ycp	2.00	Transcription
Alpha-amylase (AAM)	1smd	1.60	Hydrolase
Alpha-glucosidase (AGL)	5nn5	2.00	Hydrolase
Aldose reductase (AR)	2hv5	1.59	Oxidoreductase
dipeptidyl peptidase IV (DPP4)	2p8s	2.20	Hydrolase

## Data Availability

Data are available upon request.
